# Microfluidic 3D printing hydrogels based on fish liver decellularized extracellular matrix for liver regeneration

**DOI:** 10.1002/SMMD.20240056

**Published:** 2024-12-22

**Authors:** Haozhen Ren, Danqing Huang, Mengdi Qiu, Lingling Xue, Shaoshi Zhu, Jingjing Gan, Cheng Chen, Dayu Chen, Jinglin Wang

**Affiliations:** ^1^ Division of Hepatobiliary and Transplantation Surgery Department of General Surgery Nanjing Drum Tower Hospital The Affiliated Hospital of Nanjing University Medical School Nanjing China; ^2^ College of Medicine University of Illinois Chicago Illinois USA; ^3^ School of Pharmacy Faculty of Medicine Macau University of Science and Technology Macau China; ^4^ Department of Pharmacy Nanjing Drum Tower Hospital The Affiliated Hospital of Nanjing University Medical School Nanjing China

**Keywords:** 3D printing, decellularized extracellular matrix, hydrogel, liver regeneration, microfluidic

## Abstract

Liver tissue engineering offers potential in liver transplantation, while the development of hydrogels for scalable scaffolds incorporating natural components and effective functionalities is ongoing. Here, we propose a novel microfluidic 3D printing hydrogel derived from decellularized fish liver extracellular matrix for liver regeneration. By decellularizing fish liver and combining it with gelatin methacryloyl, the hydrogel scaffold retains essential endogenous growth factors such as collagen and glycosaminoglycans. Additionally, microfluidic‐assisted 3D printing technology enables precise modulation of the composition and architecture of hydrogels to fulfill clinical requirements. Benefiting from the natural source of materials, the hydrogels exhibit excellent biocompatibility and cellular proliferation capacity for incorporating induced pluripotent stem cell‐derived hepatocytes (iPSC‐heps). Furthermore, the macroscopic architecture and biomechanical environment of hydrogels foster optimal functional expression of iPSC‐heps. Importantly, post‐transplantation, the hydrogels significantly enhance survival rates and liver function in mice with acute liver failure, promoting liver regeneration and repair. These findings suggest that microfluidic 3D printed hydrogels represent promising candidates for liver transplantation and functional recovery.

## Graphical abstract

Microfluidic 3D bioprinting of hydrogels from fish liver dECM and GelMA enhances liver regeneration by supporting iPSC‐hep function and significantly improving survival rates in acute liver failure models. This study presents a promising approach for liver tissue engineering and regenerative medicine.

**Key points**

- 3D printed hydrogels from fish liver dECM enhance liver regeneration.
- Novel hydrogel scaffolds integrate natural bioactivity and 3D printing precision.
- Improved iPSC‐hep function and significantly higher survival rates in acute liver failure rat models.

## 1 INTRODUCTION

Liver transplantation represents a highly effective intervention for end‐stage liver diseases,^1^, ^2^, ^3^ while the scarcity of donor livers limits its broad clinical application.^4^, ^5^, ^6^ Tissue engineering, with its capacity to construct functional liver tissue, is regarded as a promising alternative to donor livers.^7^ Currently, a variety of scaffold materials have been engineered for the cultivation of hepatocytes and the facilitation of in vivo liver transplantation, thereby promoting hepatic repair.^8^, ^9^, ^10^, ^11^ Among them, hydrogels represent advanced materials that can be molded into various structures required for liver transplantation.^12^, ^13^ Despite their ability to support cellular adhesion and liver regeneration, these hydrogels often exhibit limited bioactivity and are significantly divergent from the composition of native liver tissue.^12^, ^13^, ^14^ Moreover, the majority of hydrogels necessitate intricate fabrication processes, posing potential biosafety concerns and diminishing therapeutic efficacy.^15^, ^16^ Consequently, there is an unmet need for innovative hydrogels that possess inherent bioactivity and provide adequate support for liver function.

**FIGURE 1:**
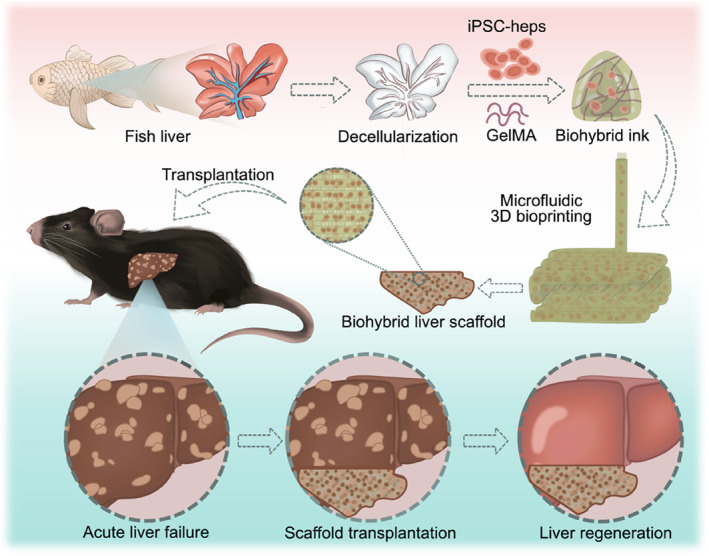
Schematic illustration of the fabrication process for microfluidic 3D printing of hydrogels based on fish liver dECM designed for hepatic regeneration.

Here, we propose a novel microfluidic three‐dimensional (3D) printing hydrogels derived from fish liver decellularized extracellular matrix (dECM) for liver regeneration, as schemed in Figure 1. The decellularized fish liver, utilized as a natural material, exhibits exceptional biocompatibility and retains a full complement of endogenous growth factors, including collagen and glycosaminoglycans.^17^, ^18^, ^19^ Moreover, compared to terrestrial animal‐derived livers, the aquatic‐derived fish liver is not only abundantly available and cost‐effective but also possesses unique functional characteristics.^20^, ^21^, ^22^ In contrast, microfluidic technology, recognized for its precise control over fluid dynamics, has been applied to fabricate diverse microfibers for drug delivery, cell culture, biosensors, and so on.^23^, ^24^, ^25^, ^26^ As an advanced manufacturing method, 3D printing facilitates the customized design of implantable hydrogels specifically tailored to address distinct clinical needs.^27^, ^28^ Leveraging these advantages, the integration of microfluidic and 3D printing technologies enables the production of customizable liver regeneration hydrogels. Therefore, the integration of fish liver decellularized hydrogel as a bioink with microfluidic‐assisted 3D printing technology to create an innovative scaffold that can effectively enhance liver repair is highly desirable.

In this study, we applied microfluidic 3D bioprinting technology to engineer a hybrid hydrogel scaffold incorporating fish decellularized liver and gelatin methacryloyl (GelMA) loaded with induced pluripotent stem cell‐derived hepatocytes (iPSC‐heps) for hepatic regeneration. Utilizing a mixture of dECM, GelMA, and iPSC‐hep as a bioink, the scaffold was fabricated through microfluidic‐assisted printing integrated with in situ photopolymerization. Owing to the high precision of microfluidic 3D printing, the hydrogels were endowed with a desirable geometric configuration and porous architecture. Besides, in situ photopolymerization effectively maintained the hydrogel structure, ensuring efficient cell encapsulation while preserving cellular bioactivity. Notably, the composite bioactive scaffold, leveraging the inherent properties of dECM and GelMA, retained ECM components, offering excellent biocompatibility and cellular adhesion. Moreover, the hydrogels' macrostructure and biomechanical milieu facilitated the robust functional expression of iPSC‐heps. Most significantly, following in vivo transplantation, the hydrogels substantially improved the survival rate and hepatic function in mice with acute liver failure (ALF), promoting liver regeneration and repair. These findings suggest that microfluidic 3D printed hydrogels possess substantial therapeutic potential within the field of regenerative medicine.

## 2 RESULTS AND DISCUSSION

**FIGURE 2:**
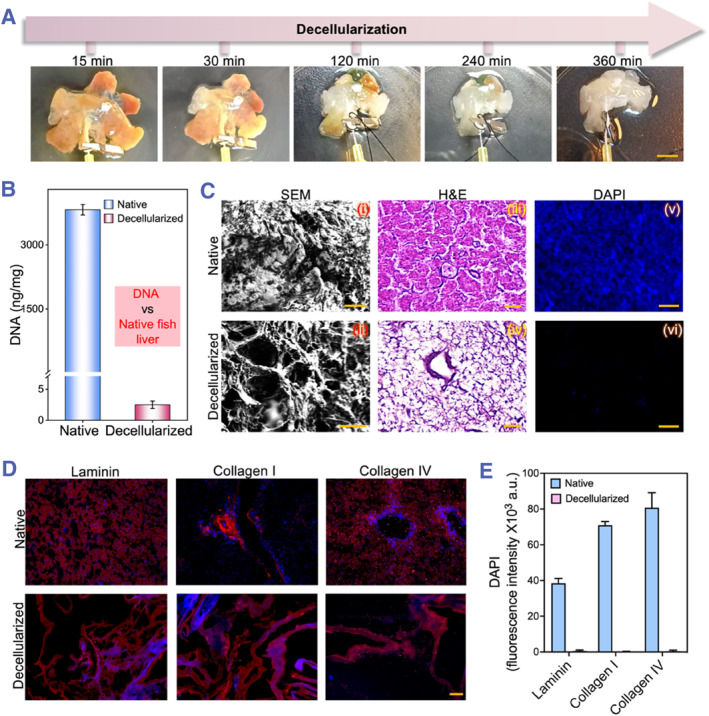
Fabrication and assessment of the fish liver dECM. (A) Temporal progression of fish liver decellularization with 1% SDS over 15, 30, 120, 240, 360 min. Scale bar represents 0.5 cm. (B) DNA content in native and decellularized fish livers. (C) SEM, H&E, and DAPI staining images of native and decellularized fish livers. Scale bars represent 100 μm in (i), 20 μm in (ii, iii, iv), and 50 μm in (v, vi). (D) Immunofluorescence staining of Laminin, Collagen I and collagen IV in native and decellularized fish livers, with nuclei visualization via DAPI staining. Scale bar is 100 μm. (E) Quantitative fluorescence analysis of DAPI in panel D, indicating the clearance of cell nuclei post‐decellularization.

In a standard experiment, 1% sodium dodecyl sulfate (SDS) was utilized as a decellularization agent and circulated through the portal vein of fish liver to facilitate the decellularization process. As perfusion time increased, the liver's overall coloration transitioned from opaque to transparent, progressing from the center to the periphery, while the intrahepatic ductal system and general structural integrity were fully preserved (Figure 2A). As demonstrated in Figure 2B, DNA quantification assays revealed that each milligram of tissue contained under 50 ng of double‐stranded DNA, indicating that the decellularization process effectively eluted DNA from the fresh tissue. In comparison to the original fish liver, scanning electron microscopy (SEM) revealed that the unique fibrous structures supporting cell adhesion and migration were retained even after decellularization (Figure 2C). Furthermore, both Hematoxylin and Eosin (H&E) staining and 4′,6‐diamidino‐2‐phenylindole (DAPI) immunofluorescence staining indicated that no significant residual nuclei were present in the decellularized liver tissues, confirming the effective removal of cells from the liver.

The decellularization process, which preserves the superior functionality of the ECM, allows us to observe under H&E staining that, despite the dissolution of hepatocyte structures, the liver's intrinsic systems remain entirely intact. This complete preservation signifies that the foundational ECM and structural scaffold, crucial for liver function, are retained, which is essential for maintaining the organ's structure and functionality. Building upon this foundation, we conducted staining for key ECM components pivotal to tissue regeneration. Our findings revealed that constituents such as Laminin, Type I Collagen, and Type IV Collagen were retained post‐decellularization (Figure 2D). Moreover, while DAPI quantification confirmed the elimination of cell nuclei (Figure 2E), the tubular structures of the liver remained intact, suggesting that the decellularization process effectively preserves the liver's vascular and biliary architectures, which are crucial for subsequent recellularization and functional restoration.

**FIGURE 3:**
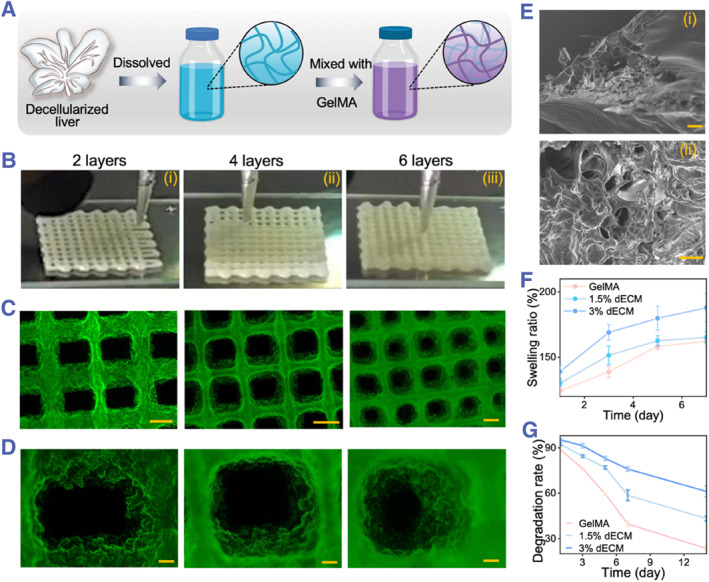
Characterization of dECM‐based hydrogel for bioprinting. (A) Fabrication of a hybrid hydrogel from dECM and GelMA for microfluidic 3D bioprinting. (B) Photographs of 2, 4, and 6‐layered scaffolds printed using microfluidic 3D bioprinting. (C, D) Fluorescent macrographs (C) and close‐ups (D) of the scaffolds after the incorporation of green fluorescent nanoparticles showcasing the 2, 4, and 6 layers. Scale bars are 500 μm in (C) and 100 μm in (D). (E) SEM images depicting the microstructure within the bioprinted scaffold, with scale bars representing 100 μm (top) and 50 μm (bottom). (F, G) Comparative analysis of swelling ratio (F) and degradation rate (G) among the non‐dECM‐based bioink group (GelMA), 1.5% dECM‐based bioink group, and 3% dECM‐based bioink group.

To harness dECM as a hydrogel scaffold for tissue engineering, we employed pepsin and acetic acid for additional digestion, while the resulting solution demonstrated low viscosity, rendering it unsuitable as a bioink. To address this issue, we contemplated incorporating GelMA, a hydrogel derived from gelatin, renowned for its exceptional biocompatibility, primarily due to its ability to support adhesion and proliferation of various cell types. Moreover, the properties of GelMA can be finely tuned to match the mechanical characteristics of diverse tissues and provide an appropriate yet adjustable viscosity conducive to the bioprinting process. Consequently, we blended dECM with GelMA to fabricate a photocurable gel ink suitable for bioprinting (Figure 3A). The 3D printing, performed under microfluidic device operation, demonstrated the smooth extrusion of the bioink from the nozzle, with rapid solidification into a shape‐retaining scaffold upon ultraviolet irradiation (Figure 3B). This gel scaffold, providing a 3D space and structure, is highly favorable for cell adhesion, proliferation, and tissue formation.

Incorporating green fluorescent nanoparticles into the bioink enabled the visualization of the dECM‐based hydrogel being printed into 2, 4, and 6 layers, with the printed structures remaining intact and increasing in thickness with each layer (Figure 3C,D). This indicates that the bioink possesses excellent photocuring properties and the capability to form multilayered structural scaffolds. SEM revealed a plethora of interconnected internal pores conducive to cell adhesion and proliferation (Figure 3E). Subsequently, we examined the water absorption capacity of dECM‐based hydrogels with varying concentrations. When the hydrogels were immersed in phosphate‐buffered saline (PBS), their overall weight gradually increased over time, demonstrating the scaffolds' hydrophilicity and water absorption capability (Figure 3F). This suggests that the scaffolds can effectively absorb surrounding culture medium and nutrients when applied to cell culture and tissue regeneration, thereby providing a favorable fluid environment for cell proliferation. Thereafter, dECM‐based hydrogels of different concentrations were placed in PBS for 14 days to assess the scaffolds' stability. As shown in Figure 3G, the 3% dECM maintained its structure more effectively, exhibiting sufficient stability for cell culture applications.

**FIGURE 4:**
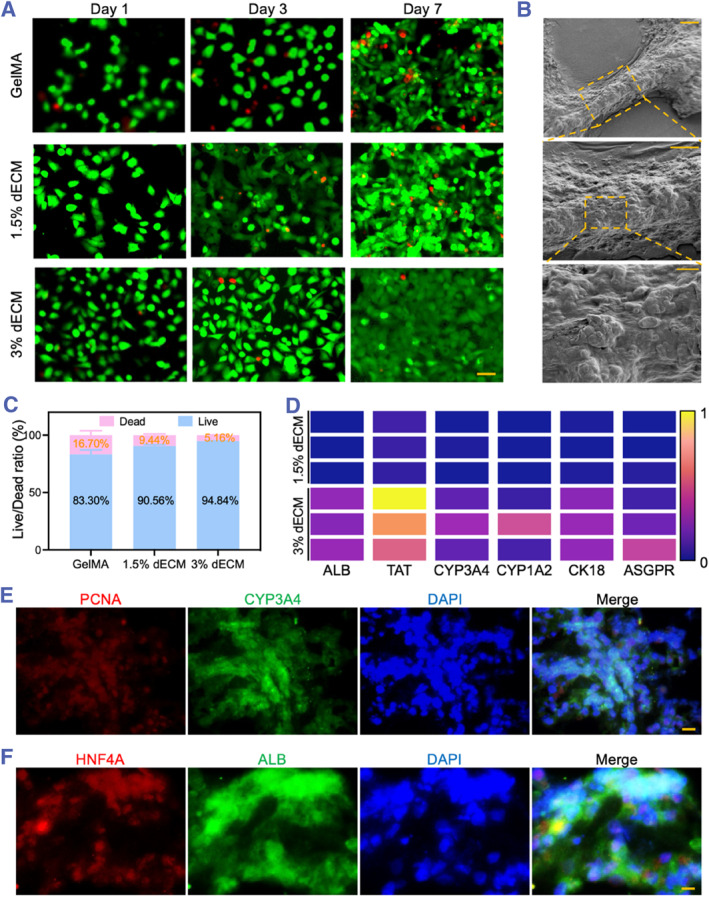
Assessment of biocompatibility and maintenance of cellular function in dECM‐based hydrogel scaffolds. (A) Live–Dead imaging of iPSC‐heps seeded on GelMA‐printed scaffolds, 1.5% dECM‐based hydrogels, and 3% dECM‐based hydrogels on different days. The scale bar is 50 μm. (B) SEM revealed the colonization of cells after 7 days of culture on a dECM‐based hydrogel scaffold. Scale bars from top to bottom are 200 μm, 100 μm, and 20 μm. (C) Quantitative analysis of Live–Dead staining for cells cultured on the three types of scaffolds after 7 days. (D) mRNA expression levels on 1.5% dECM‐based hydrogels, and 3% dECM‐based hydrogels scaffolds. (E, F) Immunofluorescent staining of cells on 3% dECM‐based hydrogels scaffolds. The scale bars are 10 μm.

To mitigate the cytotoxicity potentially resulting from residual chemicals used in the decellularization process, it is imperative to assess the biocompatibility of dECM‐based hydrogels prior to their application in tissue regeneration. We elected to utilize iPSC‐heps as a cell source for seeding on GelMA‐printed scaffolds, 1.5% dECM‐based hydrogels, and 3% dECM‐based hydrogels. Cell viability was evaluated at 1, 3, and 7 days post‐seeding using live–dead staining (Figure 4A). The findings indicated a significant increase in cell number and density over time, suggesting that all scaffold types were conducive to cell adhesion and growth. Quantitative analysis of live–dead cells on the seventh day revealed the highest cell survival rate on the 3% dECM‐based hydrogels, indicating a more favorable environment for cell viability (Figure 4C). To further appraise the efficacy of high‐concentration dECM‐based hydrogels in maintaining cellular function, we examined the secretion of albumin (ALB), CYP450 family (CYP3A4, CYP1A2) expression, and the mRNA levels of key hepatic transcription factors (TAT) and functional genes (CK18, ASGPR) in cells cultured on 1.5% and 3% dECM‐based hydrogel scaffolds (Figure 4D). The results demonstrated that a 3% dECM concentration robustly enhanced the expression of cellular functions, leading us to select 3% dECM‐based hydrogels for subsequent experiments.

To visually assess cell adhesion on the scaffold, SEM observation was conducted on the scaffolds fabricated from 3% dECM‐based hydrogels (Figure 4B). As the magnification of the SEM image progressively increased, it became evident that the cells exhibited a robust affinity for the scaffold surface. Additionally, the cellular functional expression on the scaffolds made from 3% dECM‐based hydrogels was evaluated through fluorescent staining; PCNA positivity indicated cellular proliferative capacity, while CYP3A4, ALB, and HNF4A staining confirmed the scaffold's ability to maintain hepatocyte albumin secretion, metabolic function, and biological activity (Figure 4E,F). Consequently, we deem the 3% dECM‐based hydrogel scaffolds suitable for in vivo liver regeneration applications.

**FIGURE 5:**
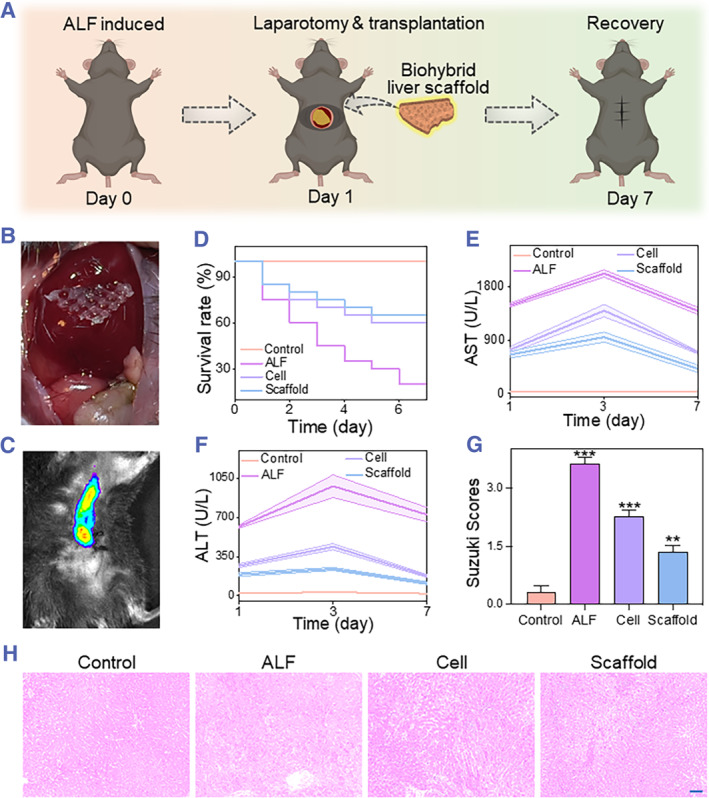
Assessment of therapeutic efficacy of hydrogel scaffold transplantation in ALF mice. (A) Schematic illustration of ALF mouse modeling and hydrogel scaffold liver transplantation. (B) Photograph of hydrogel scaffolds adhered to the liver. (C) In vivo imaging of small animals showing hydrogel scaffolds colonization on the liver. (D–F) Survival rates (D), aspartate aminotransferase (AST) (E), and alanine aminotransferase (ALT) (F) levels in the four groups of mice. (G–H) H&E staining (H) depicting the extent of liver damage and corresponding Suzuki scores (G). Scale bar in (H) represent 100 μm. ALF, acute liver failure.

Benefitting from the structure and environment of the bioprinting hydrogel conducive to the functional expression of iPSC‐heps, it has bolstered confidence in utilizing the obtained hydrogel scaffolds for the treatment of liver diseases. To induce ALF in mice, intraperitoneal injection of D‐galactose (D‐Gal) was employed. A 1 cm incision was made in the upper abdomen of the mice to expose the liver, followed by the in situ transplantation of hydrogel scaffolds to assess their efficacy in treating ALF (Figure 5A,B). To further investigate the colonization of hydrogel scaffolds within the liver, they were stained with the fluorescent cell membrane dye 1,1′‐dioctadecyl‐3,3,3′,3′‐tetramethylindocarbocyanine iodide (DIR iodide) prior to transplantation. Post‐transplantation, small animal in vivo imaging was conducted, as depicted in Figure 5C. The results indicated that the hydrogel scaffolds demonstrated a notably strong fluorescence intensity in the liver region 7 days post‐transplantation with no migration to other parts of the body.

For therapeutic efficacy analysis, 80 ALF mice were categorized into normal mice (Control group), sham‐operated controls (ALF group), hepatocyte transplantation alone (Cell group), and hydrogel scaffold transplantation (Scaffold group). Both the cell and scaffold groups received 10^7 iPSC‐heps. Survival rate monitoring over 7 days revealed that the mortality rate in the two cell‐transplanted groups was significantly lower than that in the ALF group, with the Scaffold group exhibiting the highest survival rate (Figure 5D). Additionally, markers for ALT and AST indicated more effectively controlled hepatocellular damage and promoted liver function recovery in the Scaffold group (Figure 5E,F). H&E staining of liver samples revealed extensive necrosis in the ALF group; however, in both cell therapy groups, the necrotic areas were significantly reduced, particularly in the Scaffold group, where hepatic necrosis was minimal, resulting in the lowest Suzuki score (Figure 5G,H). This suggested that liver damage was restored after Scaffold transplantation.

**FIGURE 6:**
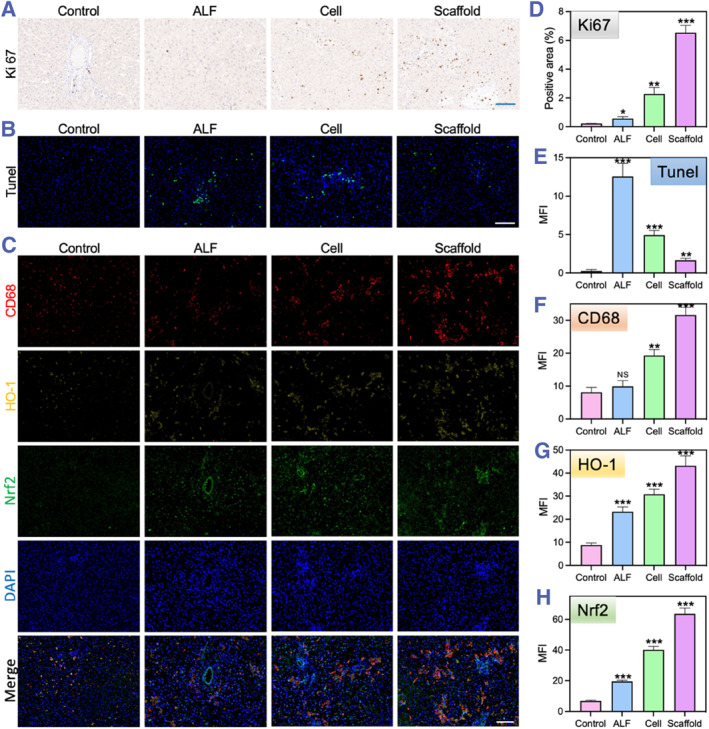
Immunohistochemical evaluation of the therapeutic effects of scaffold transplantation. (A–C) Representative Ki67 (A), TUNEL (B), and merged fluorescence images of CD68, Nrf2, HO‐1, and DAPI (C) from liver samples of different groups. (D–H) Quantitative fluorescence analysis of Ki‐67 (D), TUNEL (E), CD68 (F), Nrf2 (G), and HO‐1 (H). The scale bars measure 100 μm.

Furthermore, we employed Ki67 staining to assess the proliferation of hepatocytes post‐transplantation. Compared to the ALF group, both the Cell group and Scaffold group exhibited significantly improved hepatocyte proliferation, with the Scaffold group demonstrating a more pronounced effect on cell proliferation (Figure 6A,D). Similarly, TUNEL staining revealed a marked reduction in apoptotic levels of hepatocytes in the Cell group and Scaffold group, with the Scaffold group showing an even lower number of apoptotic cells (Figure 6B,E). Given that inflammatory damage and oxidative stress responses are also major factors affecting liver regeneration, to explore the specific role of the Scaffold group in liver repair, we performed CD68, HO‐1, and Nrf2 staining on liver tissues (Figure 6C,F–H). The results indicated that compared with the ALF and Cell groups, the Scaffold group displayed higher CD68 expression, suggesting greater macrophage aggregation and resistance to inflammatory responses. Additionally, the activation of the Nrf2/HO‐1 antioxidant pathway in the Scaffold group also indicated that it could promote liver recovery by enhancing antioxidant capacity. Therefore, we believe that the Scaffold group's transplant treatment may facilitate liver repair through anti‐inflammatory and antioxidant effects.

## 3 CONCLUSION

In conclusion, our study presented a novel approach to liver regeneration through the fabrication of microfluidic 3D bioprinted hydrogels derived from fish liver dECM. The integration of dECM with GelMA resulted in a bioink that demonstrated superior biocompatibility and supported the functional expression of iPSC‐heps. This combination has been underexplored in the literature, and our work provides valuable insights into the synergistic effects of these components on hepatocyte function and liver regeneration. The precision of microfluidic 3D printing technology enabled the creation of hydrogel scaffolds with tailored geometric configurations and porous architectures, which are essential for effective cell adhesion, proliferation, and tissue formation. In vivo transplantation of these scaffolds into mice with ALF significantly improved survival rates and hepatic function, promoting liver regeneration and repair. The therapeutic efficacy of the hydrogels was further substantiated by immunohistochemical evaluations, which revealed enhanced hepatocyte proliferation, reduced apoptosis, and increased resistance to inflammatory and oxidative stress responses. These findings underscore the potential of microfluidic 3D bioprinting hydrogels as a promising modality for liver transplantation and functional recovery, offering a viable alternative to traditional liver transplantation methods and holding significant promise for the regenerative medicine.

## 4 MATERIALS AND METHODS

### 4.1 Decellularization of fish liver tissue

Utilize fresh fish to meticulously extract a complete liver with intact vasculature. Then, venous cannulation was employed to continuously perfuse the liver with 1% SDS (10 mL/min flow rate) at ambient temperature for decellularization. Subsequently, the liver was rigorously washed with PBS to eliminate any residual decellularizing agents. Perform all procedures under sterile conditions. Finally, 1% pepsin and 100 mM acetic acid was employed to solubilize the dECM.

### 4.2 Fabrication of hydrogel scaffold based on dECM through microfluidic 3D printing

After disinfecting the microfluidic printing device, a 3D scaffold model was designed for printing. The prepared dECM at concentrations of 1.5% or 3% was thoroughly mixed with GelMA (7.5%) and a photoinitiator to form a bioink that solidifies immediately under ultraviolet irradiation.

### 4.3 DNA content

Perform DAPI nuclear staining on tissue sections of dECM to assess nuclear residue. Concurrently, DNA content analysis of dECM was conducted using a DNA quantification kit, and a spectrophotometer, expressing DNA concentration as nanograms per milligram of dry tissue weight.

### 4.4 Extraction and detection of cellular mRNA

Total cellular RNA was extracted utilizing Trizol reagent followed by reverse transcription into complementary DNA (cDNA) employing the Takara PrimeScript RT Master Mix kit. Quantification of target mRNA transcripts was performed via real‐time SYBR Green RT‐PCR. We used *β*‐actin as the endogenous control, and applied the 2^−ΔΔCt^ method for normalizing the gene of interest genes' relative mRNA expression levels.

### 4.5 Animal experiments

Eighty C57/B6‐L mice were randomized into the control, ALF, Cell, and scaffold groups, with 20 mice per group. An ALF model was induced according to established methods, and the ALF, Cell, and Scaffold groups received intraperitoneal administration of D‐Gal (0.6 g/kg). The control group was left untreated. The ALF, Cell, and Scaffold groups were then administered an in situ injection of PBS, a hepatocyte suspension (with cell numbers equivalent to the Scaffold group), and the Scaffold, respectively. All mice survived the laparotomy. Over the subsequent 7 days, daily survival rates were recorded, and serum levels of ALT and AST were measured on days 1, 3, and 7. After a 7‐day treatment period, livers from each group were harvested for H&E, Ki67, TUNEL, CD68, Nrf2, and HO‐1 staining.

### 4.6 Statistical analysis

All test values are presented as mean ± standard deviation. Student's *t*‐test was employed to assess differences between the two groups, with *p* < 0.05 indicating statistical significance. Survival curves were constructed utilizing the Kaplan–Meier estimation and SPSS version 18.0 software.

## AUTHOR CONTRIBUTIONS

Jinglin Wang and Danqing Huang conceptualized the idea and structured the article; Jingjing Gan and Shaoshi Zhu conducted literature reviews and composed the manuscript; Mengdi Qiu, Lingling Xue, Cheng Chen, Dayu Chen and Haozhen Ren contributed to the writing process; and Jinglin Wang and Haozhen Ren oversaw the manuscript.

## CONFLICT OF INTEREST STATEMENT

The authors declare that there are no competing interests.

## ETHICS STATEMENT

This study strictly followed the guidelines set by the Animal Ethics Committee of the Drum Tower Hospital affiliated with the Medical School of Nanjing University (No. 20230401).
